# Supplementary material for: Cryo-EM snapshots of a native lysate provide structural insights into a metabolon-embedded transacetylase reaction
Source: Nat Commun. 2021 Nov 26;12:6933. doi: 10.1038/s41467-021-27287-4 (PMC8626477; doi:10.1038/s41467-021-27287-4)
Supplement: Supplementary file 1 — Supplementary Information [file 41467_2021_27287_MOESM1_ESM.pdf]

Supplementary information

CryoEM snapshots of a native lysate provide structural insights into a metabolon-embedded transacetylase reaction

Christian Tüting[1#], Fotis L. Kyrilis[1,2#], Johannes Müller[2], Marija Sorokina[2,3,4], Ioannis Skalidis[1,2], Farzad Hamdi[1], Yashar Sadian[5], Panagiotis L. Kastritis[1,2,6,*]

[1]Interdisciplinary Research Center HALOmem, Charles Tanford Protein Center, Martin Luther University Halle-Wittenberg, Kurt-Mothes-Straße 3a, Halle/Saale, Germany.
[2]Institute of Biochemistry and Biotechnology, Martin Luther University Halle-Wittenberg, Kurt-Mothes-Straße 3, Halle/Saale, Germany.
[3] RGCC International GmbH, Baarerstrasse 95, Zug, 6300, Switzerland
[4] BioSolutions GmbH Weinbergweg 22, 06120, Halle/Saale, Germany
[5]Bioimaging Center, Université de Genève, Sciences II, 1211, Genève 4, Switzerland
[6]Biozentrum, Martin Luther University Halle-Wittenberg, Weinbergweg 22, Halle/Saale, Germany.

#contributed equally
*Correspondence: panagiotis.kastritis@bct.uni-halle.de (PLK)

Supplementary Tables

Supplementary Table 1. Sequences of recombinant proteins used in this study and their corresponding molecular weight (Da)

Protein	Recombinant Sequence	Molecular weight [Da]
E1α	Met-E1$\alpha_{37\text{-}411}$-His$_6$	42530.25
E1b	Met-E1$\beta_{7\text{-}382}$-His$_6$	41587.86
E2	Met-E2$_{29\text{-}459}$-His$_6$	46415.84
E3BP	Met-E3BP$_{34\text{-}442}$-His$_6$	44390.15
E3	Met-E3$_{35\text{-}504}$-His$_6$	51341.91

Supplementary Table 2. **CryoEM data collection, refinement and validation statistics**

	#1 name (EMDB-13066) (PDB 7OTT)
**Data collection and processing**	
Magnification	92000 X
Voltage (kV)	200 kV
Electron exposure (e–/Å^2)	30
Defocus range (μm)	-0.5 to -1.5
Pixel size (Å)	1.5678
Symmetry imposed	I
Initial particle images (no.)	296779
Final particle images (no.)	10249
Map resolution (Å)	3.84
FSC threshold	0.143
Map resolution range (Å)	3.5-5.0
**Refinement**	
Initial model used (PDB code)	7BGJ
Model resolution (Å)	3.85
FSC threshold	0.143
Model resolution range (Å)	4.039-3.9
Map sharpening *B* factor (Å^2)	-94.5985
Model composition	
Non-hydrogen atoms	1583
Protein residues	209
Ligands	n/a
B factors (Å^2)	
Protein	22.55
Ligand	n/a
R.m.s. deviations	
Bond lengths (Å)	0.003
Bond angles (°)	0.72
Validation	
MolProbity score	1.75
Clashscore	11.80
Poor rotamers (%)	0.00
Ramachandran plot	
Favored (%)	97.10
Allowed (%)	2.90
Disallowed (%)	0.00

Supplementary Figures

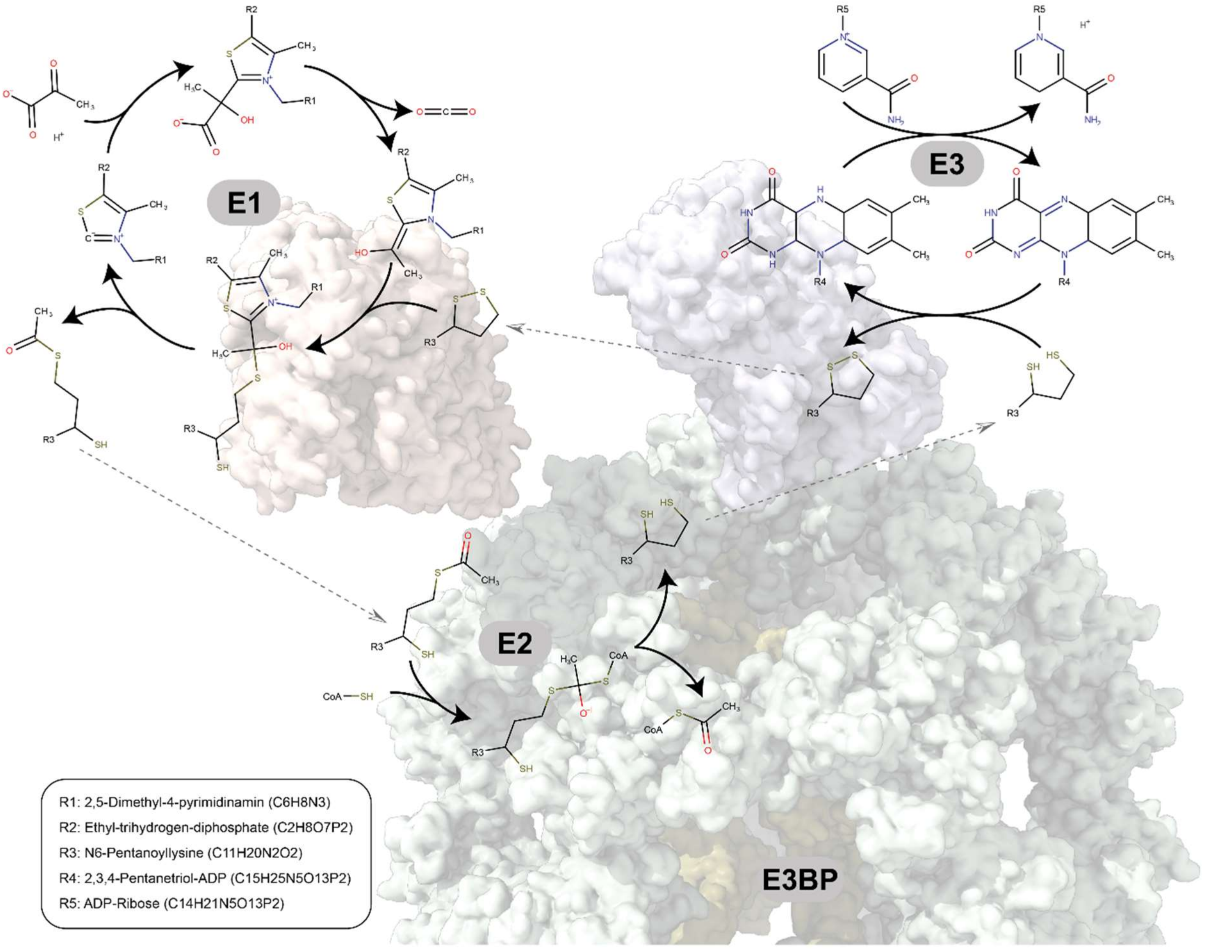

Supplementary Figure 1. Detailed reaction schema of the pyruvate oxidation, catalyzed by the fungal PDHc metabolon. The conversion of pyruvate to acetyl-CoA, which enters the Krebs Cycle, is catalyzed by three distinct active sites at the E1, E2, and E3 subunits. E3BP does not have a catalytically active site. E1 is tethered to the core by the E2, while E3BP binds E3. The reaction requires the three steps in a distinct order: (1) At the active site of E1, Pyruvate gets decarboxylated, and the acetyl moiety is transferred onto an LD present in the N-ter regions of the E2 or the E3BP. (2) At the active site of the E2 core, the acetyl moiety is transferred onto a CoA molecule, and the lipoate in the LD is fully reduced. (3) The lipoate gets reoxidized at the E3 active site, where NADH + H^+ is produced.

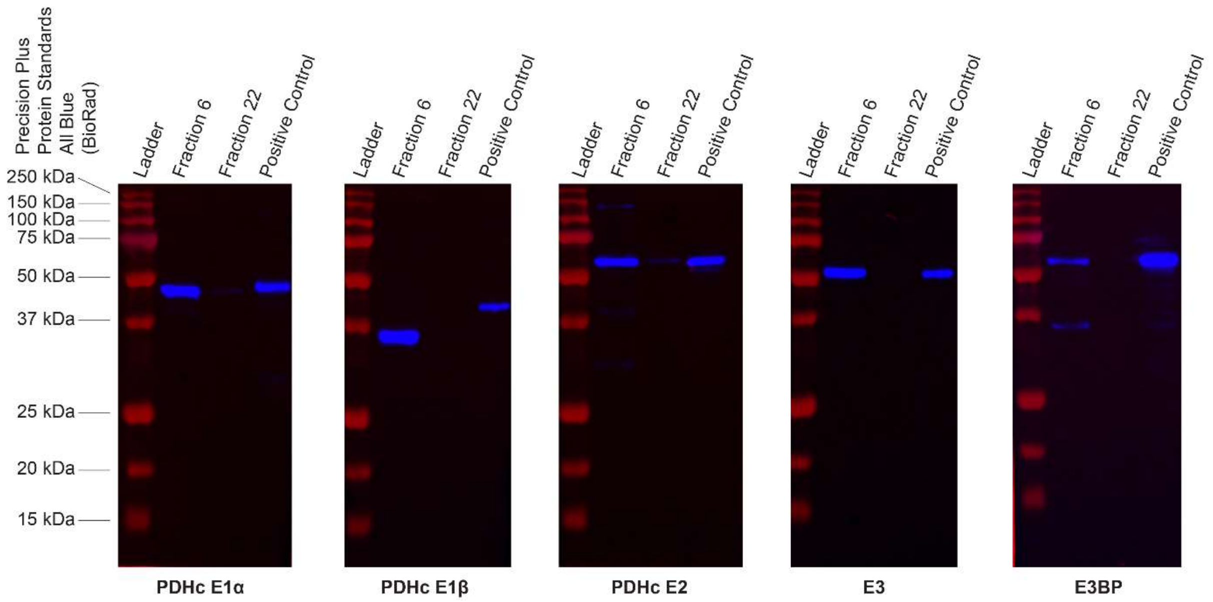

Supplementary Figure 2. Uncropped images of the western blots, identifying all components of the PDHc metabolon (n = 1). All proteins of the PDHc metabolon are present in the high molecular-weight fraction 6, but not in the later fraction 22 (negative control). Recombinant proteins, used for antibody production were used as positive control. The apparent shift in molecular weight for PDHc E1β might be explained by either higher charge to mass ratio in the native protein by phosphorylation, or by a significant difference of the native protein in comparison to the recombinant protein due to signal-peptide cleavage on the native protein and the affinity tag on the recombinant protein.

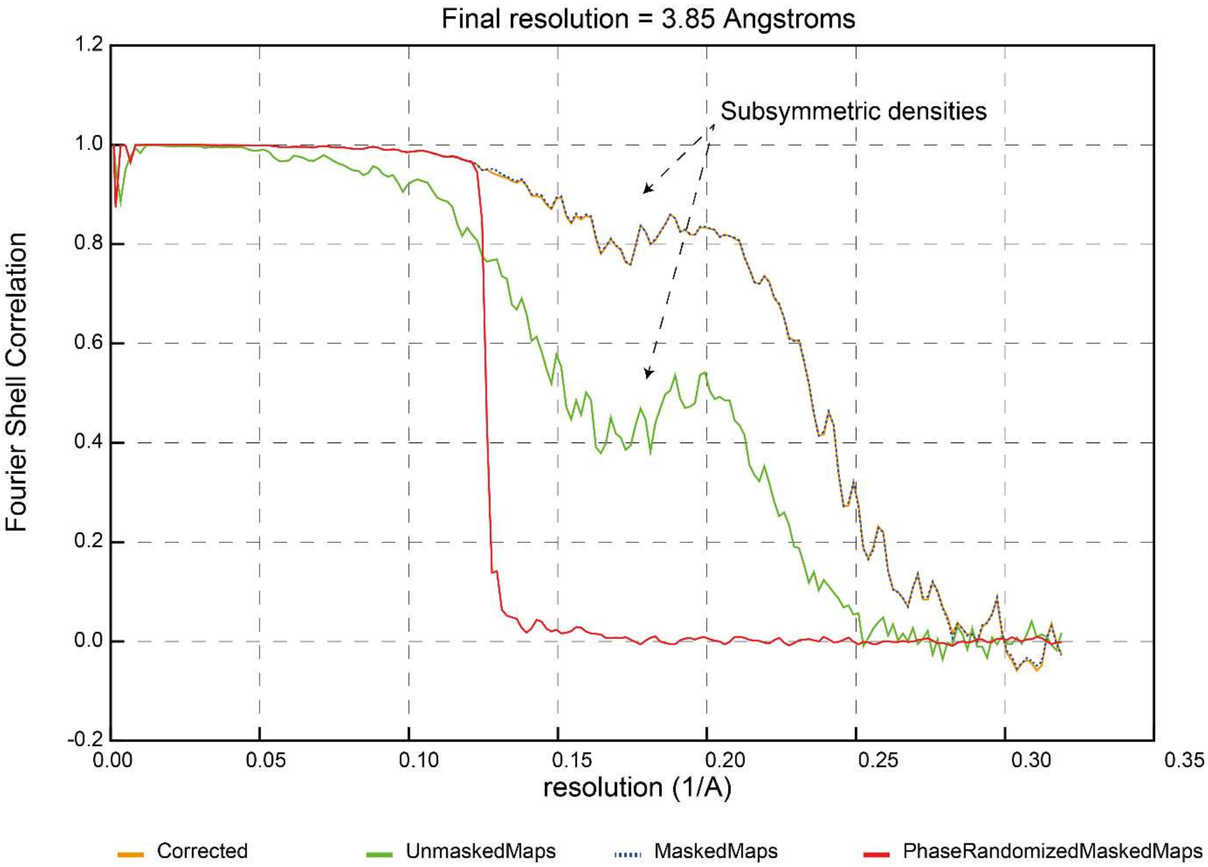

Supplementary Figure 3. Fourier Shell Correlation of the icosahedrally-averaged cryoEM map corresponding to PDHc core. The dip in the FSC curve indicates the presence of additional, subsymmetric densities, including E3BP (with tetrahedral symmetry) and external E1/E3 subunit (asymmetric).

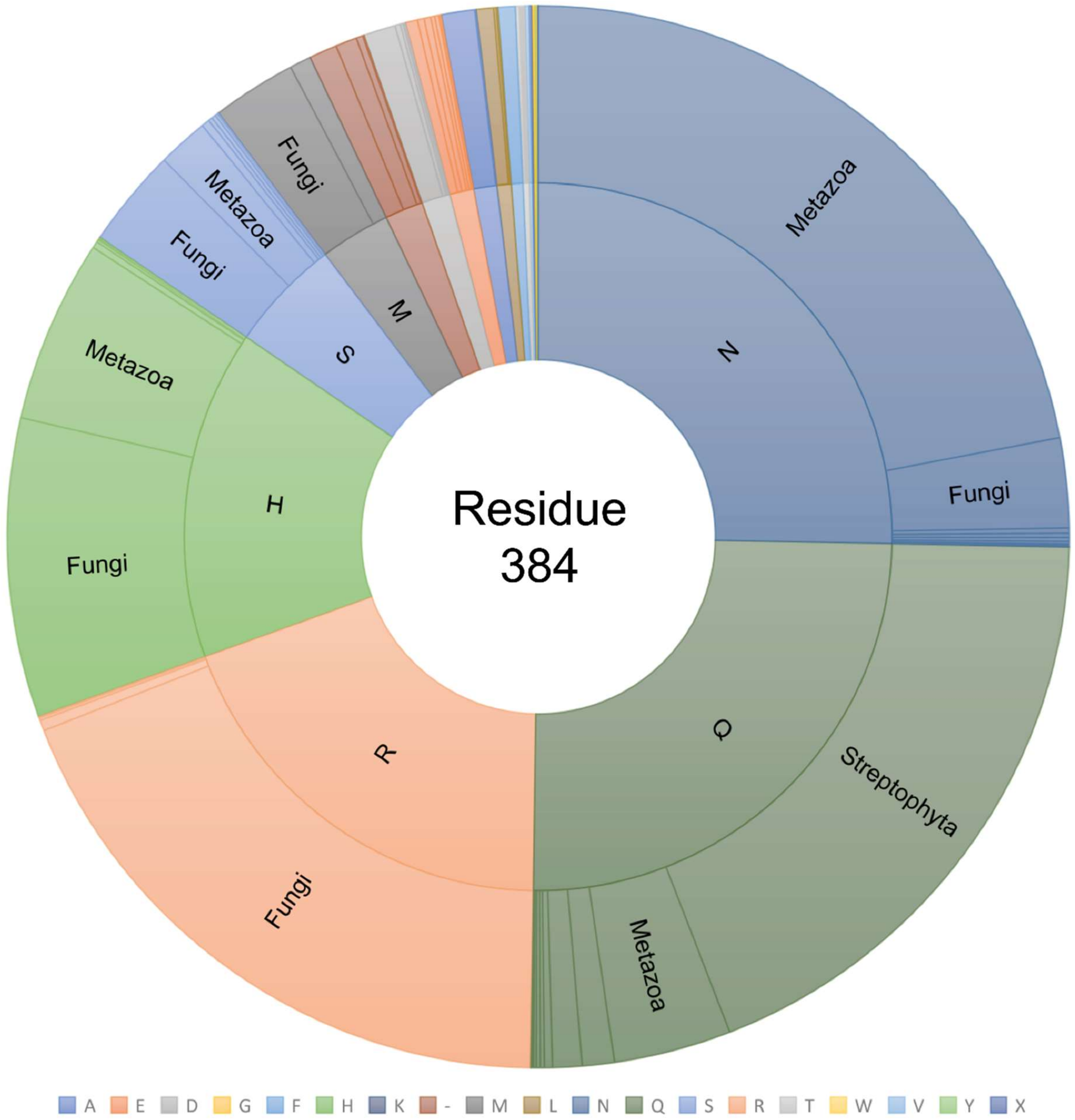

Supplementary Figure 4. Sunburst plot showing the conservation rate for residue 384. The conservation rate was calculated by aligning 2460 unique UniProt entries, derived by blastp identification to be similar to selected PDHc E2 proteins. Inner ring displays the residue at position 384, second ring the eukaryotic kingdom.

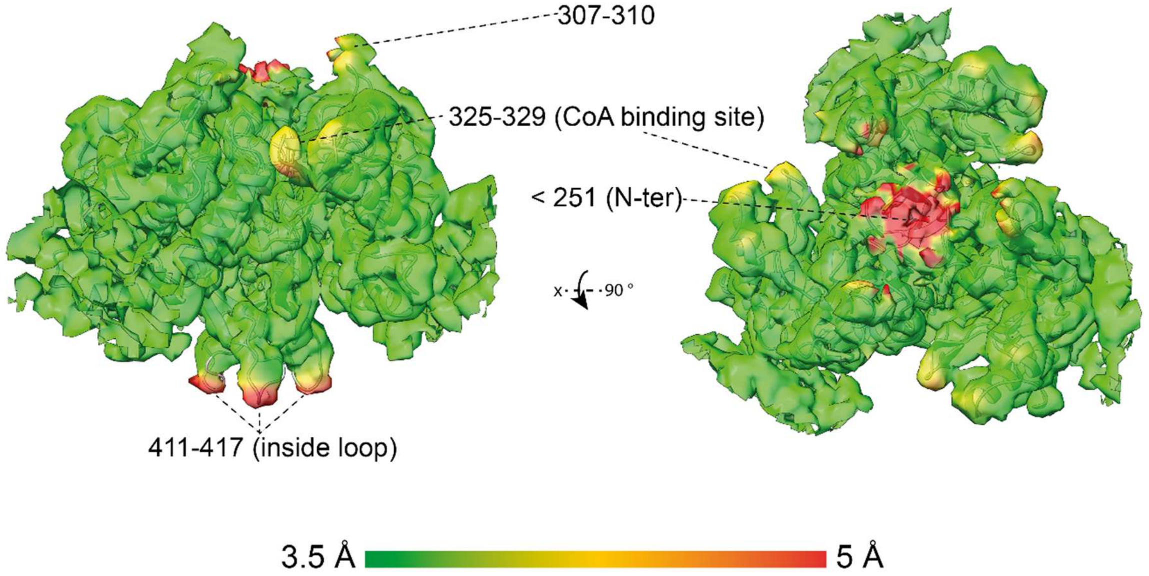

Supplementary Figure 5. Local resolution map for the PDHc core. The density map corresponding to an E2 trimer is displayed. Local resolution was calculated with cryoSPARC and density was colored with ChimeraX. The reconstruction shows a very uniform resolution, except the highlighted regions: (a) the *N-ter* of the E2, where the flexible linker is located, and a nearby loop region (307-310) (b) a loop-region (325-329) near the CoA binding site, and (c) the inside loop region (411-417).

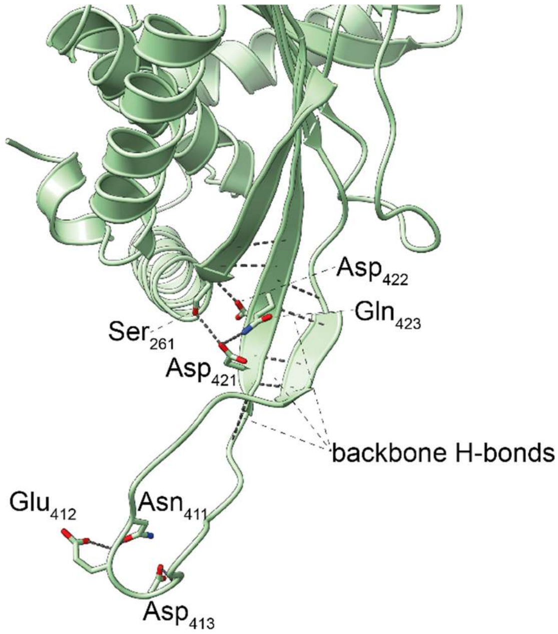

Supplementary Figure 6. A zoom into the E2 protein highlights the internal loop and its anchor-induced stabilization. The inside loop is stabilized by an extended hydrogen bonding network. Residues involved with their side chains are displayed as licorice and labelled accordingly, while residues involved only with their backbone atoms are not displayed.

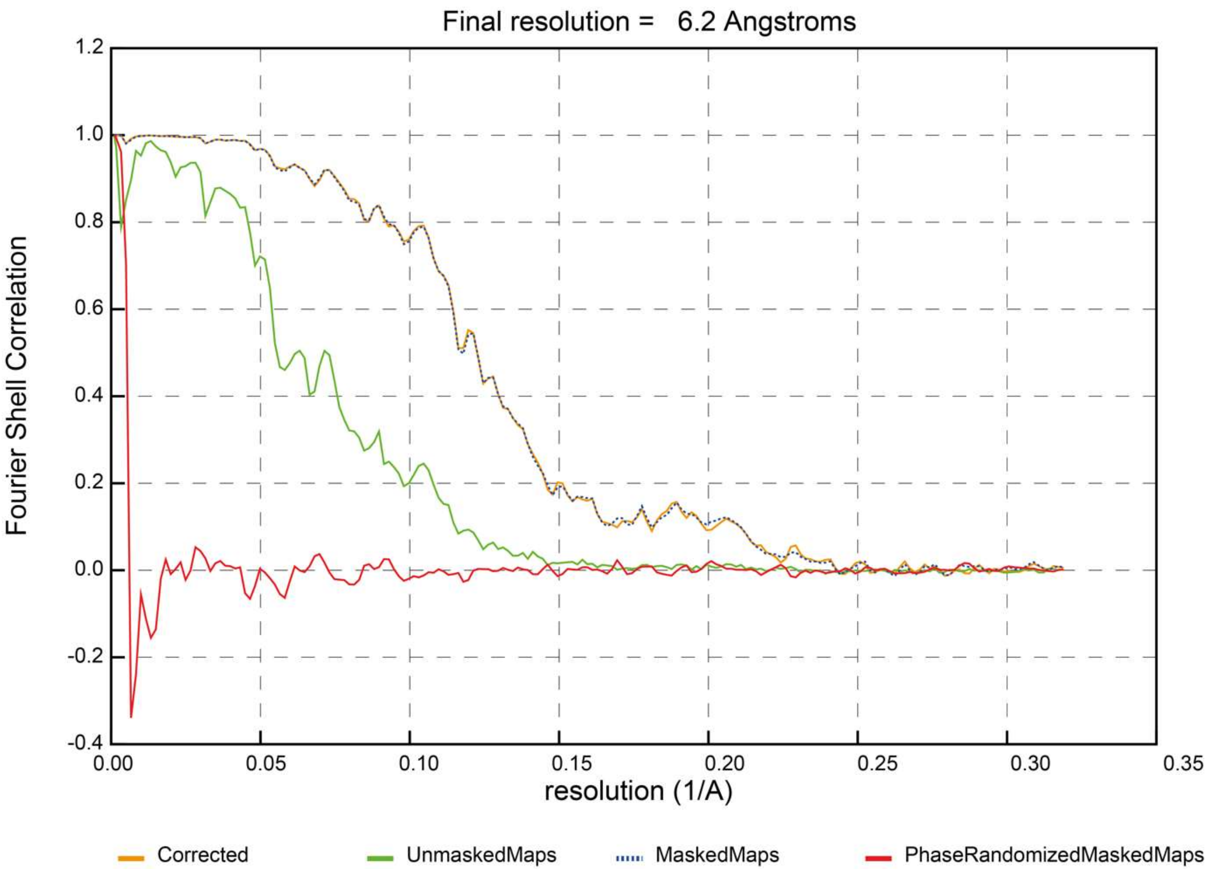

Supplementary Figure 7. Fourier Shell Correlation of the C2-averaged cryoEM map corresponding to PDHc core scaffold.

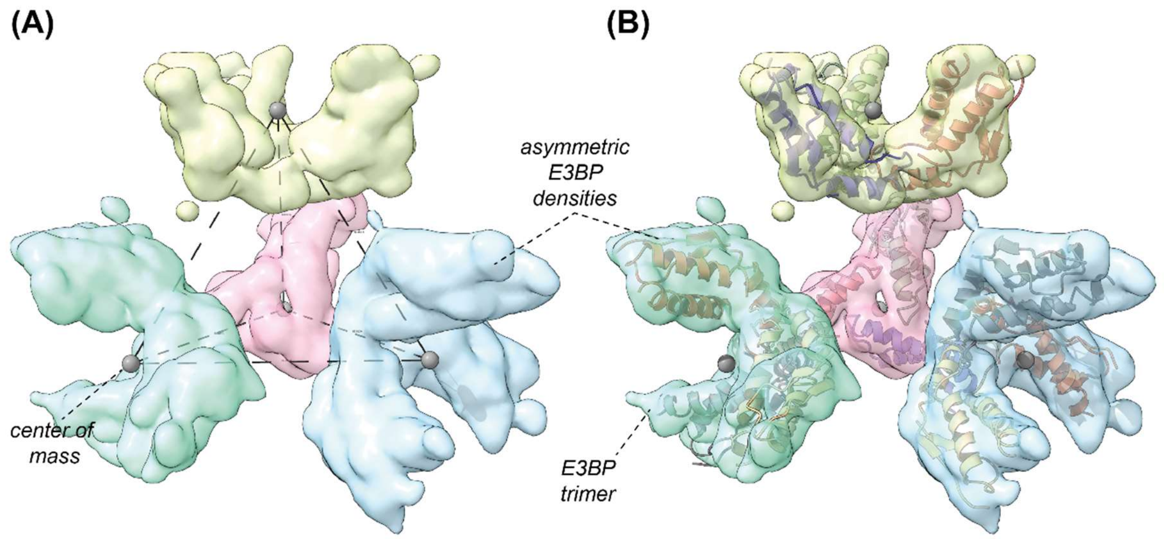

Supplementary Figure 8. Tetrahedral symmetry of trimeric E3BP. (A) Isolated E3BP density of the C1 reconstructed cryoEM map. The center of mass of each trimer forming a tetrahedron with a distance of each 70 Å. (B) In each density, a E3BP trimer is located. Map is displayed at a contour level of 0.03.

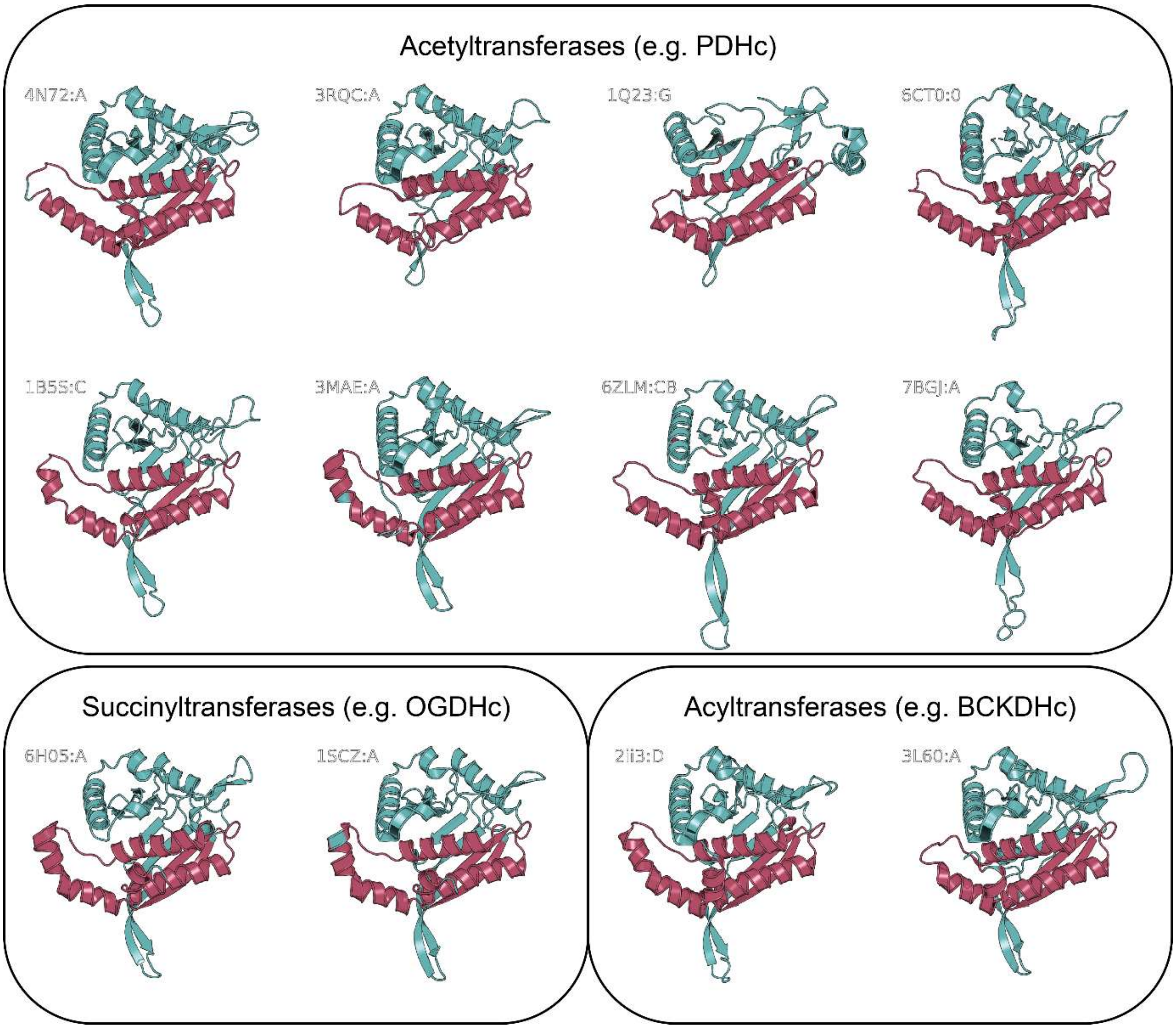

Supplementary Figure 9. Structure-based conservation of the minimal fold discovered for E3BP across transferases. The minimal fold of E3BP, consisting of a two-standed β-sheet with three helical elements is found in various other acyltransferases with different higher-order assemblies. Acetyltransferases usually form icosahedral assemblies, whereas succinyl- and acyltransferases cubic assemblies. The PDB-IDs and corresponding polypeptide chains of each molecule is annotated; the discovered minimal fold is highlighted with brick color.

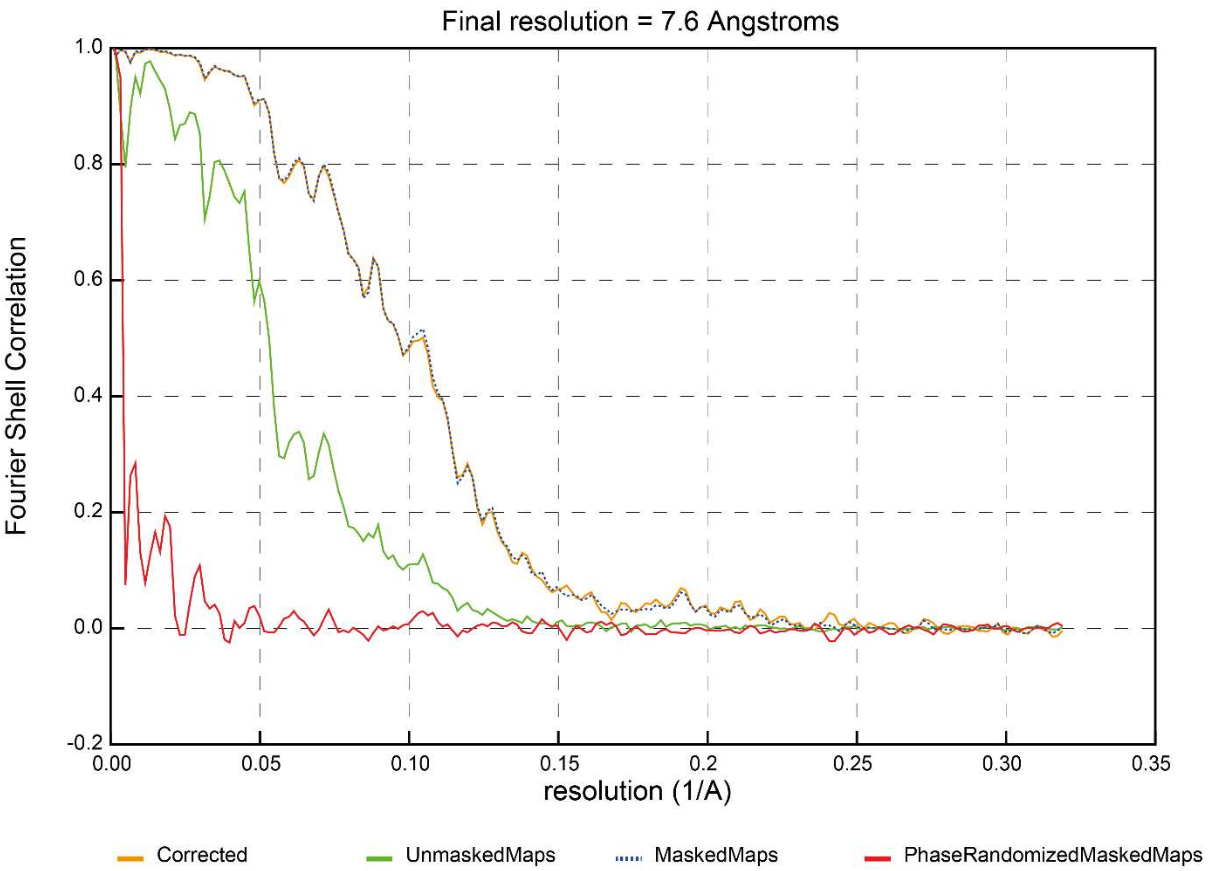

Supplementary Figure 10. Fourier Shell Correlation of the asymmetrically reconstructed cryoEM map corresponding to the PDHc core scaffold.

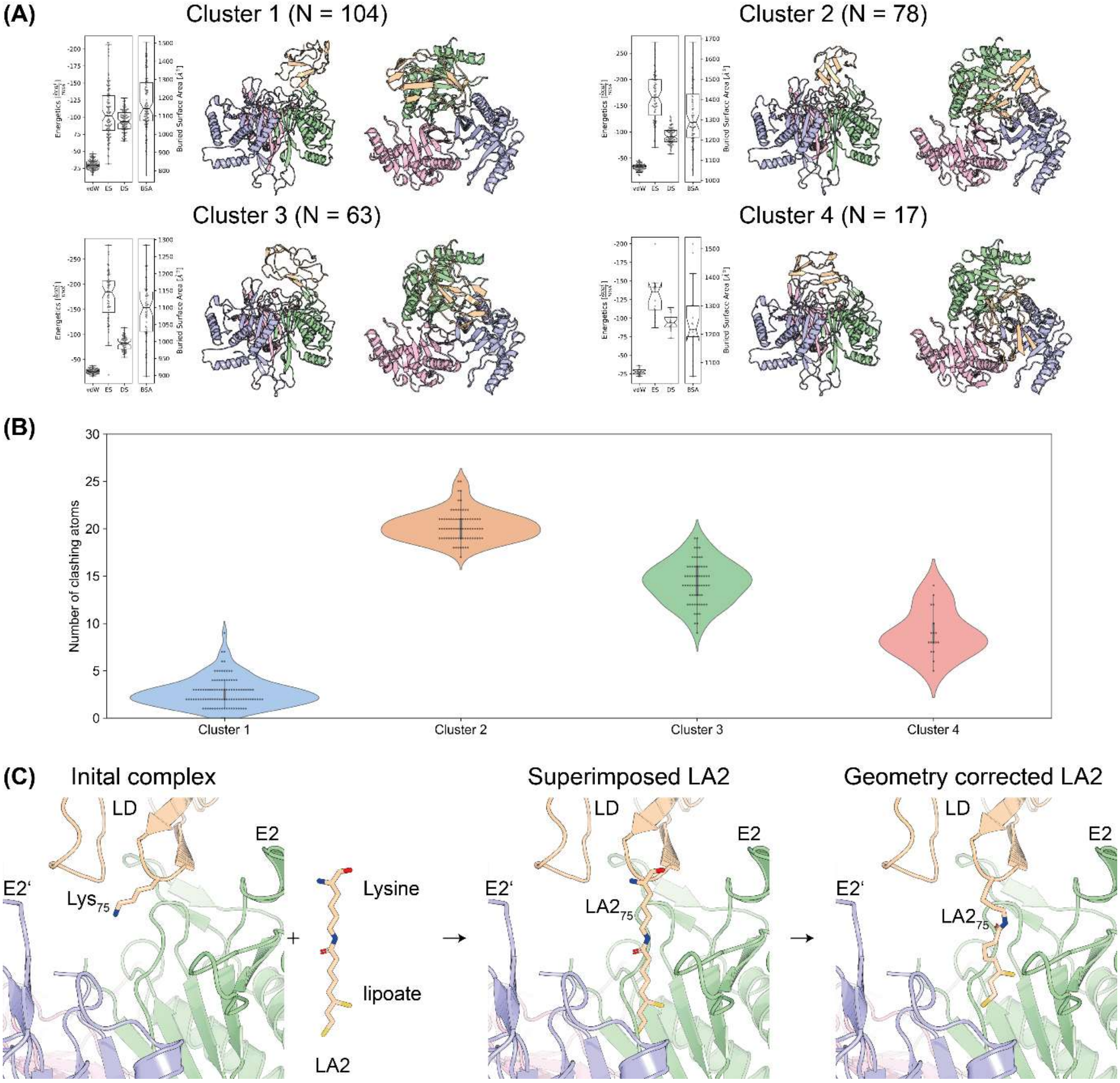

Supplementary Figure 11. Docking analysis of LD and E2, and modeling of the active site. (A) Overview showing all formed clusters with more than 15 models during HADDOCK-based biomolecular docking. The cluster size (N) is given in the header while calculated energetics are shown as bar plots. A top and a side view of a representative structure from each cluster is shown after alignment. The E2 trimer is colored in magenta, pink and green, while the LD in wheat color. The box minima represent the 25th percentile, the box maxima the 75th percentile, the Notch indicated the data's median, whiskers extend to the minimum and maximum value inside of a 1.5 interquartile range. All datapoints are overlayed as beeswarm plot. (B) Statistically analysis of the lipoate binding pocket. In a radius of 1.5 Å around the Euclidian distance between the Cα of Lys75 in the LD and CoA binding site, all atoms were counted for each model in each cluster. The box minima inside the violin plot represents the 25th percentile, the maxima the 75th percentile, whiskers extend to the minimum and maximum value inside of a 1.5 interquartile range. (C) Workflow corresponding to the modelling of the lipoate modification for the Lys75 of the LD. The representative structure of cluster 1 was superimposed with the chemical structure of lipoyllysine (PDB Ligand ID LA2). The pdb file was manually curated. The rigid structure of LA2 was geometrically corrected by using the Coot function "*Regularize*

Zone". The $LA2_{75}$ fitted the binding pocket without clashes while maintain all residue localizations in its binding site.

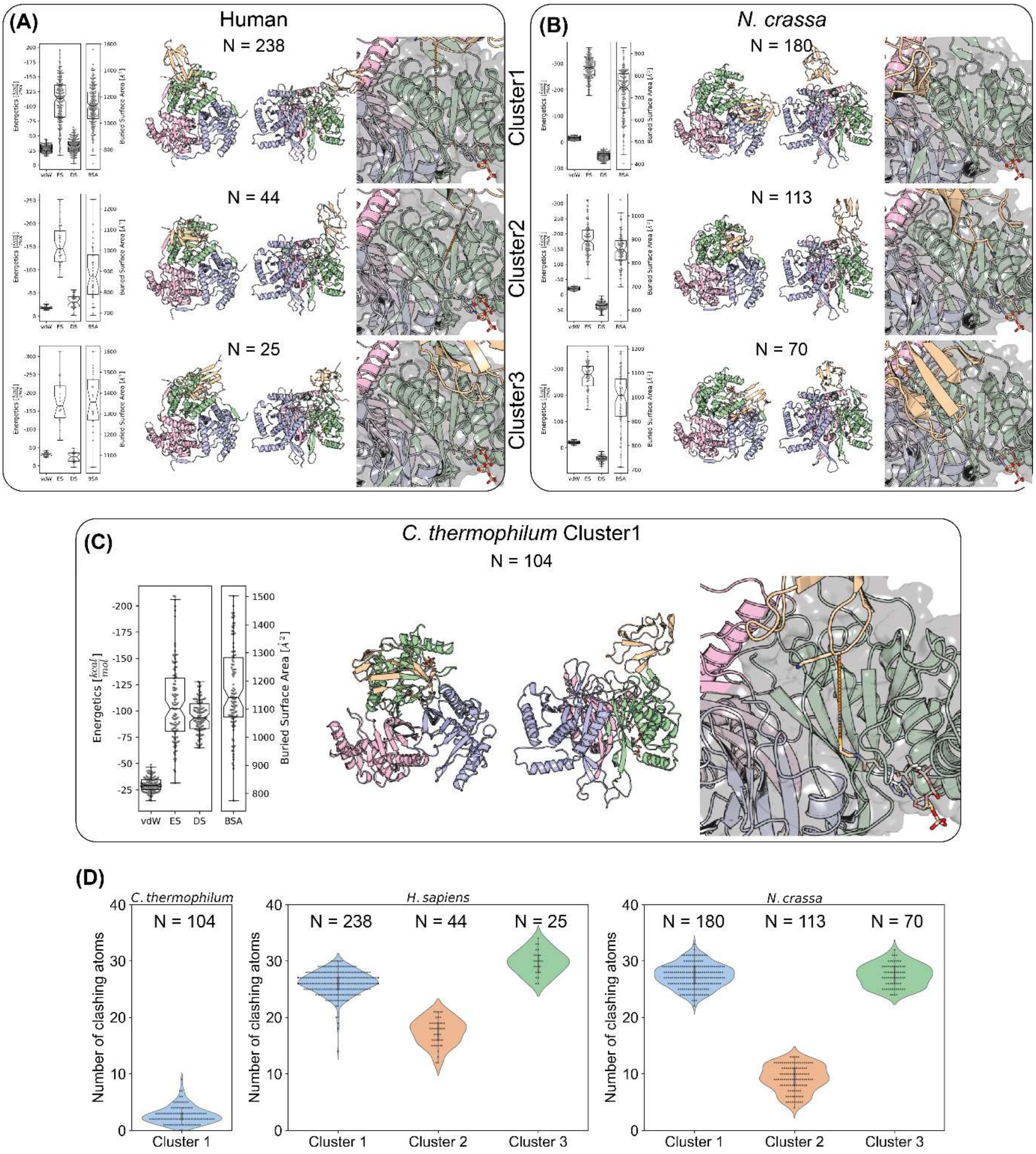

Supplementary Figure 12. Docking of the LD to the human and *N. crassa* E2 core. (A/B) The Top 3 clusters of both docking calculations contain 61 % (human) and 73 % (*N. crassa*) of all water refined models. The box minima represent the 25th percentile, the box maxima the 75th percentile, the Notch indicated the data's median, whiskers extend to the minimum and maximum value inside of a 1.5 interquartile range. All datapoints are overlayed as beeswarm plot. (C) The Top1 cluster of the docking calculation of the Chaetomium proteins is shown as direct comparison. Energetics of each cluster are given as bar plots and the top-scoring docked structure is shown from the top and side views. The E2 trimer is colored in magenta, pink and green, while the LD in wheat color. The surface of the E2 is shown in grey and the Euclidean distance calculated between the lipoyllysine of the respective LD and the thiol group of a superimposed CoA is shown as dashed line. (D) The number of atoms around this distance in a radius of 1.5 Å are counted and displayed as violin plots. The

box minima inside the violin plot represents the 25th percentile, the maxima the 75th percentile, whiskers extend to the minimum and maximum value inside of a 1.5 interquartile range.
Only in the *C. thermophilum* complex the lipoate binding pocket is accessible highlighted by the interatomic distance not disturbed by the E2 N-ter backfolded structural element which is present in the human and *N. crassa* E2s.

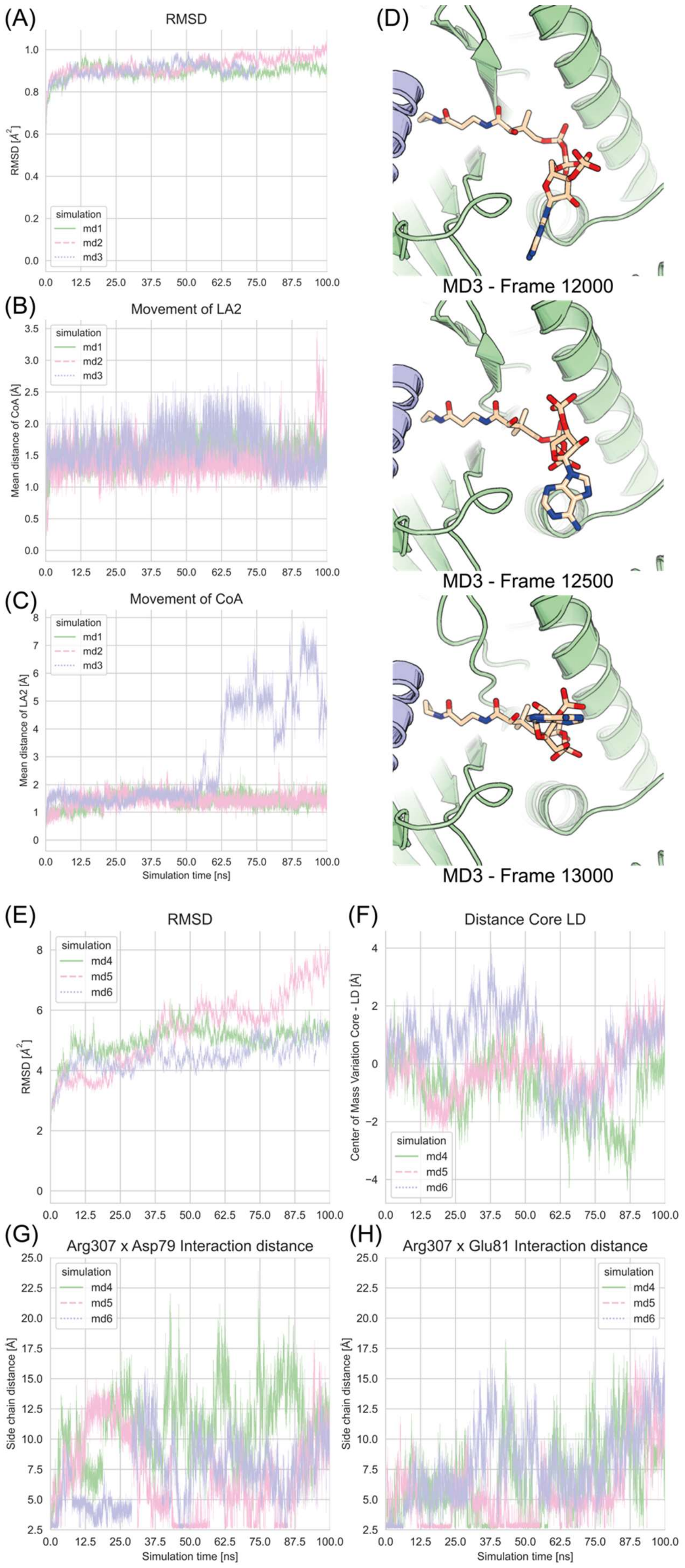

(A)
RMSD
RMSD [Å²]
simulation
md1
md2
md3
(B)
Movement of LA2
Mean distance of CoA [Å]
(C)
Movement of CoA
Mean distance of LA2 [Å]
Simulation time [ns]
(D)
MD3 - Frame 12000
MD3 - Frame 12500
MD3 - Frame 13000
(E)
RMSD
md4
md5
md6
(F)
Distance Core LD
Center of Mass Variation Core - LD [Å]
(G)
Arg307 x Asp79 Interaction distance
Side chain distance [Å]
(H)
Arg307 x Glu81 Interaction distance

Supplementary Figure 13. Detailed analysis of the performed MD-Simulations. (A) “Hard-restrained MD” triplicate across 100 ns – RMSD plot. The plot shows overall stability for each MD simulation. (B) LA2 from initial frame was used as a reference to calculate mean distance of its atoms across the simulation. The plot shows fast relaxation and stable localization over time. (C) Same as (B), but for CoA. In one of the three simulations, after 60 ns, flexibility is observed. (D) Explanation of the observed flexibility in (C); The CoA adenosine moiety undergoes conformational rearrangements, escaping the charge complementarity imposed by its binding site. This observation possibly shows an initial mechanism for its release. (E) “Soft-restrained” MD triplicate across 100 ns – RMSD plot. The plot shows increased flexibility due to the applied protocol while 2/3 simulations show relative stability. MD5, after ~75 ns shows increased flexibility due to loop movements in the unbound E2 chain. (F) Relative distance of the calculated center of mass (COM) between the LD and the bound E2 normalized to the initial frame calculated from the “soft-restrained” MD simulations. The COMs remain in relative proximity across the replicates. (G-H) Distance between the electropositive and electronegative atoms of Arg307 and Asp79/Glu81 present on the bound E2 core and the LD, respectively. Distances were calculated from the “soft restrained” MD simulations. These residues form an ionic interaction predicted by docking; The MD simulations show that the ionic interaction reoccurs across the simulation and across replicates; More frequent ionic interactions are observed between Glu81 and Arg307 due to the larger flexibility and longer side-chain of the glutamate residue.
