# Supplementary material for: Cryo-EM snapshots of a native lysate provide structural insights into a metabolon-embedded transacetylase reaction
Source: Nat Commun. 2021 Nov 26;12:6933. doi: 10.1038/s41467-021-27287-4 (PMC8626477; doi:10.1038/s41467-021-27287-4)
Supplement: Supplementary file 3 — Reporting Summary [file 41467_2021_27287_MOESM3_ESM.pdf]

nature research

Corresponding author(s): Panagiotis L. Kastritis

Last updated by author(s): 05/11/2021

Reporting Summary

Nature Research wishes to improve the reproducibility of the work that we publish. This form provides structure for consistency and transparency in reporting. For further information on Nature Research policies, see Authors & Referees and the Editorial Policy Checklist .

Statistics

For all statistical analyses, confirm that the following items are present in the figure legend, table legend, main text, or Methods section.

n/a	Confirmed	
☐	☒	The exact sample size (*n*) for each experimental group/condition, given as a discrete number and unit of measurement
☐	☒	A statement on whether measurements were taken from distinct samples or whether the same sample was measured repeatedly
☐	☒	The statistical test(s) used AND whether they are one- or two-sided<br>*Only common tests should be described solely by name; describe more complex techniques in the Methods section.*
☐	☒	A description of all covariates tested
☐	☒	A description of any assumptions or corrections, such as tests of normality and adjustment for multiple comparisons
☐	☒	A full description of the statistical parameters including central tendency (e.g. means) or other basic estimates (e.g. regression coefficient) AND variation (e.g. standard deviation) or associated estimates of uncertainty (e.g. confidence intervals)
☐	☒	For null hypothesis testing, the test statistic (e.g. *F*, *t*, *r*) with confidence intervals, effect sizes, degrees of freedom and *P* value noted<br>*Give P values as exact values whenever suitable.*
☒	☐	For Bayesian analysis, information on the choice of priors and Markov chain Monte Carlo settings
☒	☐	For hierarchical and complex designs, identification of the appropriate level for tests and full reporting of outcomes
☒	☐	Estimates of effect sizes (e.g. Cohen's *d*, Pearson's *r*), indicating how they were calculated

Our web collection on statistics for biologists contains articles on many of the points above.

Software and code

Policy information about availability of computer code

Data collection	Cryo-EM data were collected with the EPU package installed on the Glacios microscope. The data collection strategy is listed in the Methods section of the manuscript as well as all software used including their versions.
Data analysis	The data collection strategy and the analysis is listed in the Methods section of the manuscript as well as all software. Detailed versions include: (a) Data processing: RELION 3.1, cryoSPARC 3.2; (b) Data visualization: PyMOL 2.4, ChimeraX 1.2; (c) Structure analysis: Clustal Omega, Xplor-NIH 3.3; (d) Modeling: MODELLER 9.12, COOT 0.9.6, NAMD 2.13, HADDOCK 2.2; (e) MD software: NAMD 2.13 with default version of SHAKE algorithm, Linux-x86_64-multicore-CUDA 45; (f) Charmm-Gui: CHARMM General Force Field (CGenFF) program version 2.4.0

For manuscripts utilizing custom algorithms or software that are central to the research but not yet described in published literature, software must be made available to editors/reviewers. We strongly encourage code deposition in a community repository (e.g. GitHub). See the Nature Research guidelines for submitting code & software for further information.

Data

Policy information about availability of data

All manuscripts must include a data availability statement. This statement should provide the following information, where applicable:

- Accession codes, unique identifiers, or web links for publicly available datasets
- A list of figures that have associated raw data
- A description of any restrictions on data availability

We used data from publicly available databases. This includes 4 entries in PDB (6CT0, 6ZLM, 7BGJ, 1EAD). Source data for displayed plots are provided with this paper. The 3D maps are available at EMDB database (accession code EMD-13066), the molecular model of E2 at the PDB database (accession code PDB ID: 7OTT) and the computational models as well as the parameter files for the MD simulation at the SBGrid database (accession code 848). All accessions will be freely available for downloading after revision.

Field-specific reporting

Please select the one below that is the best fit for your research. If you are not sure, read the appropriate sections before making your selection.

- [x] Life sciences
- [ ] Behavioural & social sciences
- [ ] Ecological, evolutionary & environmental sciences

For a reference copy of the document with all sections, see nature.com/documents/nr-reporting-summary-flat.pdf

Life sciences study design

All studies must disclose on these points even when the disclosure is negative.

Sample size	2810 cryo-EM images were acquired from Chaetomium thermophilum fractionated cell extracts using the EPU software and in our experience was sufficient to yield high-resolution cryo-EM maps from recorded movies. This sample of thousand of images is sufficient for high-resolution cryoEM image analysis as shown throughout the manuscript.
Data exclusions	CryoEM data were processed in Relion, which excluded low-quality data to reach high-resolution using statistical methods. The exclusion criteria are pre-established as implemented in Relion, which is a common practice in cryo-EM.
Replication	We developed a complete pipeline described in the Materials and Methods allowing for the reproducibility of the experiments and analysis.
Randomization	No randomization is relevant for this study because it is not relevant for this type of structural biology research. Cryo-EM data, when collected, are random because they are acquired under low-dose, so the researcher never knows how the acquired image would look like.
Blinding	Blinding is not relevant for this type of study performed here. Large parts of image processing and structure calculation and analysis are based on computer automation. In addition, only one type of input data are used (single-particles) - therefore, no risk of bias exists.

Reporting for specific materials, systems and methods

We require information from authors about some types of materials, experimental systems and methods used in many studies. Here, indicate whether each material, system or method listed is relevant to your study. If you are not sure if a list item applies to your research, read the appropriate section before selecting a response.

Materials & experimental systems

n/a	Involved in the study	
[ ]	[x]	Antibodies
[x]	[ ]	Eukaryotic cell lines
[x]	[ ]	Palaeontology
[x]	[ ]	Animals and other organisms
[x]	[ ]	Human research participants
[x]	[ ]	Clinical data

Methods

n/a	Involved in the study	
[x]	[ ]	ChIP-seq
[x]	[ ]	Flow cytometry
[x]	[ ]	MRI-based neuroimaging

Antibodies

Antibodies used

1. Name: Rabbit polyclonal antibody a-E1α,
Cat#: U9576EL020-28,
RRID: AB_2888984,
Investigator: Dr. Panagiotis L. Kastritis - Martin Luther University Halle-Wittenberg,
Supplier name: Genscript
Concentration of Stock: 0.506 mg/ml
Concentration used for the blottings: 0.2 μg/ml
Host strain: New Zealand Rabbit
* Kyrilis FL, Semchonok DA, Skalidis I, Tüting C, Hamdi F, O'Reilly FJ, Rappsilber J, Kastritis PL. Integrative structure of a 10-megadalton eukaryotic pyruvate dehydrogenase complex from native cell extracts. Cell Rep. 2021 Feb 9;34(6):108727. doi: 10.1016/j.celrep.2021.108727. PMID: 33567276.

2. Name: Rabbit polyclonal antibody a-E1β,
Cat#: U842VFI110-94,
RRID: AB_2893234,
Investigator: Dr. Panagiotis L. Kastritis - Martin Luther University Halle-Wittenberg,
Supplier name: Genscript
Concentration of Stock: 3.32 mg/ml

Concentration used for the blottings: 0.2 µg/ml
Host strain: New Zealand Rabbit

3. Name: Rabbit polyclonal antibody a-E2p,
Cat#: U9576EL020-18,
RRID: AB_2888985,
Investigator: Dr. Panagiotis L. Kastritis - Martin Luther University Halle-Wittenberg,
Supplier name: Genscript
Concentration of Stock: 1.03 mg/ml
Concentration used for the blottings: 0.2 µg/ml
Host strain: New Zealand Rabbit
* Kyrilis FL, Semchonok DA, Skalidis I, Tüting C, Hamdi F, O'Reilly FJ, Rappsilber J, Kastritis PL. Integrative structure of a 10-megadalton eukaryotic pyruvate dehydrogenase complex from native cell extracts. Cell Rep. 2021 Feb 9;34(6):108727. doi: 10.1016/j.celrep.2021.108727. PMID: 33567276.

4. Name: Rabbit polyclonal antibody a-E3,
Cat#: U842VFI110-9,
RRID: AB_2893235,
Investigator: Dr. Panagiotis L. Kastritis - Martin Luther University Halle-Wittenberg,
Supplier name: Genscript
Concentration of Stock: 0.973 mg/ml
Concentration used for the blottings: 0.2 µg/ml
Host strain: New Zealand Rabbit

5. Name: Rabbit polyclonal antibody a-E3BP,
Cat#: U842VFI110-18,
RRID: AB_2893236,
Investigator: Dr. Panagiotis L. Kastritis - Martin Luther University Halle-Wittenberg,
Supplier name: Genscript
Concentration of Stock: 1.023 mg/ml
Concentration used for the blottings: 0.2 µg/ml
Host strain: New Zealand Rabbit

*the ones with the asterisk are the ones that have been used in the mentioned manuscript as well

All Ab were in purified by antigen affinity in Phosphate Buffered Saline (PBS, pH 7.4) including 0.02% Sodium Azide as preservative. Concentration was measured by NanoDrop Spectrophotometer A280nm and purity by SDS-PAGE.

Supplier name: Abcam
Goat Anti-Rabbit IgG H&L (HRP) ab 205718
Type: Polyclonal
Storage buffer: pH: 7.40 Preservative: 0.1% Proclin 300 Solution Constituents: PBS, 1% BSA, 30% Glycerol (glycerin, glycerine)
Quality control: The antibody used for conjugation reacts with rabbit immunoglobulins of all classes. Cross-reactions as determined by ELISA for the unconjugated antibody (ab182016): Mouse IgG, rat IgG, and chicken IgY, less than 2%. Human IgG, less than 7%.
Purification: Immunogen affinity purified
Purification notes: This antibody was isolated by affinity chromatography using antigen coupled to agarose beads and conjugated to Horse Radish Peroxidase (HRP).
Host species: Goat
Target species: Rabbit
Stock concentration: 2 mg/ml
Dilution used in the experiments: 0.1 µg/ml

Validation

All the antibodies described above have been verified by the manufacturer and supplier, Genscript and two of them (#1 and #3 in the list were also previously extensively tested (see publication reported under the section "antibodies used"). Our negative control experiment validated the specificity of the antibodies, and the recombinant proteins, overexpressed and purified served as positive controls. All the species specificity, noisy signaling, and application were also validated with positive and negative controls. Controls were also performed to ensure that there was no crosstalk between channels of imaging.
